# Baicalin protects against renal interstitial fibrosis in mice by inhibiting the TGF-β/Smad signalling pathway

**DOI:** 10.1080/13880209.2022.2097700

**Published:** 2022-08-07

**Authors:** Hui Wang, Qingtao Jiang, Lizhu Zhang

**Affiliations:** a Department of Clinical Medicine, Jiangsu Health Vocational College, Nanjing, China; b Department of Nanxin Pharm, Nanjing, China

**Keywords:** Fibroblasts, Smad2, Smad3, UUO

## Abstract

**Context:**

Baicalin, a flavonoid extracted from radix scutellariae, possesses various pharmacological effects, including protective effects on renal interstitial fibrosis (RIF), but its possible role and mechanisms have not been fully elucidated.

**Objective:**

This study explores the protective effects and mechanisms of baicalin on RIF.

**Materials and methods:**

C57BL/6 male mice were divided into six groups: sham, model, low baicalin, middle baicalin, high baicalin and positive drug groups. The unilateral ureteral obstruction (UUO) model of RIF was constructed and treated with baicalin doses (10, 20 and 40 mg/kg) and a positive control drug (valsartan, 8 mg/kg). H&E staining was used to observe the pathological changes in renal tissues, Masson staining was performed to evaluate collagen deposition in renal tissues, and immunohistochemical examination was adopted to determine α-SMA and extracellular matrix (ECM) expression. Primary mouse fibroblasts were isolated, extracted and treated with baicalin and/or TGF-β. qRT-PCR and enzyme-linked immunosorbent assay (ELISA) were applied to detect the inflammatory responses. Moreover, ECM and TGF-β/Smad expression levels were evaluated by western blot assay.

**Results:**

Baicalin ameliorated RIF in UUO mice by inhibiting fibrosis and inflammatory responses. The TGF-β/Smad pathway was significantly suppressed in the UUO mouse model. Additionally, baicalin significantly inhibited ECM expression and inflammatory factors in fibroblasts treated with TGF-β. TGF-β/Smad pathway activation was significantly decreased in fibroblasts.

**Discussion and conclusions:**

These findings support the use of baicalin as a potential therapeutic option for the treatment of RIF by possibly inhibiting the TGF-β/Smad signalling pathway.

## Introduction

Renal interstitial fibrosis (RIF) is a common pathological manifestation of all kinds of chronic kidney diseases (CKDs) that develop to the point of end-stage renal failure (Waasdorp et al. 2019). There are many causes of CKD, including primary glomerulonephritis, secondary glomerulonephritis, hypertensive nephropathy, diabetic nephropathy, hereditary nephropathy and renal tubulointerstitial disease (Deng et al. 2017; Barutta et al. 2018). International epidemiological survey data show that CKD is the most common chronic disease in developed countries, and the prevalence rate in the adult population has reached 10.2–13.0% (Murphy et al. 2016). According to the epidemiological survey of CDK in China, the prevalence rate of CKD in adults over 18 years old has reached 10.8% (Xi et al. 2013). Moreover, approximately 2% of CKD patients will develop end-stage renal failure, which requires kidney transplantation or dialysis to maintain their lives (Lv et al. 2018).

The occurrence and development of RIF are affected by many factors (Tanino et al. 2016). Regardless of the aetiology, the most important pathogenesis and pathological features of RIF are an imbalance of extracellular matrix (ECM) synthesis and/or degradation, and excessive deposition in normal renal interstitium and renal tubules (Song et al. 2017; Jiang et al. 2020). The inflammation, induced by many types of kidney diseases as a protective response, activates immune cells and intrinsic renal cells to release profibrotic cytokines and growth factors that lead to fibrosis, causing renal disease to deteriorate into the end stage (Meng et al. 2014). Thus, the control of ECM-related proteins and inflammatory factors suggests the ability to ameliorate RIF. Increasing evidence suggests that TGF-β1 associates with the pathogenesis of renal fibrosis by regulating the transdifferentiation of tubular epithelial cells into myofibroblasts. The TGF-β1/Smad pathway has essential impact on renal fibrosis, and Smad2 and Smad3 are two crucial downstream mediators in response to the biological effects of TGF-β1 (Xu J et al. 2018).

Despite the rapid development of medicine, new technologies and drugs have been developed, but there is no specific drug to inhibit or reverse RIF (Hamed 2019). In recent years, many researchers in China have focussed on the exploration of traditional Chinese medicine (TCM), and it has been discovered that TCM influences the development of RIF via multiple targets and multiple pathways (Wang V et al. 2016). Baicalin is an active ingredient monomer of radix scutellariae with antibacterial, anti-inflammatory, antioxidant, antiviral and anti-allergic effects (Yan et al. 2016) and has been confirmed to be closely related to RIF. For example, baicalin inhibits RIF formation and development by reducing TGF-β1 expression (Zheng et al. 2017). In addition, baicalein improves tubulointerstitial fibrosis by inducing apoptosis of myofibroblasts by regulating the PI3K/Akt pathway (Cai et al. 2017). In addition, total aglycone extract of radix scutellariae improves renal fibrosis induced by mercuric chloride in rats (Liu et al. 2016). The study of whether TCM plays an antifibrotic role through the TGF-β/Smad signalling pathway provides a theoretical basis for the anti-RIF effects of TCM and further demonstrates the close relationship between the TGF-β/Smad signalling pathway and RIF. Therefore, this study aimed to observe the effects of baicalin on RIF in unilateral ureteral obstruction (UUO) mice and fibroblasts, and to explore the effects of baicalin on ECM synthesis, inflammation, and the TGF-β/Smad pathway to provide a new experimental basis for baicalin in RIF treatment.

## Materials and methods

### Animals

Thirty-six C57BL/6 male mice (6 weeks old) were purchased from Shanghai Laboratory Animals Center (Shanghai, China). All mice were reared at a constant temperature (22 ± 2 °C), maintained at 30–50% humidity, and kept in a 12 h day–night light cycle. Mice were kept sterile and had free access to food and water. This study was approved by the Ethics Committee of Jiangsu Health Vocational College with approval number NXLL-007-(A) and was performed in accordance with the principles of the Declaration of Helsinki.

### RIF model in mice

Mice were randomly divided into six groups, including sham operation (sham), model (UUO), low baicalin, middle baicalin, high baicalin and positive drug (valsartan), with six mice in each group. In addition to the sham group, mice in each group were anaesthetized with pentobarbital sodium, and UUO mouse models were established by left ureteral ligation according to a previous study (Bersani-Amado et al. 2016). Baicalin (HPLC ≥98%, batch number: SB8020, Solarbio Life Sciences, Beijing, China) was administered at concentrations of 10, 20 and 40 mg/kg (Li et al. 2015), while valsartan was administered at a concentration of 8 mg/kg. The sham group and UUO group were given an equal volume of distilled water by gavage once a day for a total of 14 days.

### Isolation of kidney and blood samples

Kidney isolation was performed as described in a previous study (Skrypnyk et al. 2013). In brief, the dorsal skin along the midline of the mouse (approximately 1.5 cm) was cut by scissors and forceps. Then, the skin and subcutaneous layers over the left and right dorsal sides through this incision by blunt dissection were separated by scissors and forceps. A small incision was made through the left flank muscle and fascia above the kidney and the left kidney was exteriorized. Then, the upper and lower poles of the kidney were dissected free from surrounding tissue and washed three times with 0.01 M PBS (10010023, Thermo Fisher Scientific, Boston, MA). Blood was collected from mice by eyeball extraction to assess inflammatory cytokines in serum.

### Isolation and culture of primary mouse fibroblasts

Collagenase digestion and screen separation assays were used to culture mouse fibroblasts. The connective tissues and cortex of the kidney surface were cut off under aseptic conditions, and then the medullary tissues were cut into chylous shapes after washing with PBS. Then, 0.1% collagenase III (3.5 times volume, LS004206, Seebio, Shanghai, China) was added and digested at 37 °C for 10 min. The supernatant was passed through 80 mesh sieves, and the suspension under 80 mesh sieves was collected in serum-free medium. The collected suspension was centrifuged and the supernatant was discarded. Serum-free culture medium was added to the suspension. The suspension was inoculated into a cell culture bottle at 10^5^ cells/mL, and incubated in a 5% CO_2_ incubator at 37 °C (Ding et al. 2017).

All experiments were divided into six groups: including the blank group, TGF-β1 group treated with 3 ng/mL TGF-β1 (R&D Systems, Minneapolis, MN), baicalin groups (50, 100 and 150 μmol/L) and baicalin (150 μmol/L)+TGF-β/Smad activator (10 μmol/mL; SRI-011381 hydrochloride; Sigma-Aldrich, St. Louis, MO). After three days, the cells were collected for further analysis.

### H&E staining assay

Renal tissues were fixed for 24 h with 4% paraformaldehyde (P395744, Aladdin, Shanghai, China), and then cut into 4 μm slices. The haematoxylin–eosin staining kit was purchased from Solarbio Life Sciences (Beijing, China, G1120). The slices were stained for 5 min with haematoxylin. Afterwards, the slices were differentiated for 50 s with differentiation solution, and restained for 2 min with eosin according to the kit’s instructions. Finally, the slices were photographed to observe pathological changes using a light microscope (Nikon, Minato, Japan, ×200 magnification). HE staining was used to evaluate the degree of renal interstitial injury according to related literature (Hu et al. 2017).

### Masson staining assay

Masson's trichrome stain kit was purchased from Solarbio Life Sciences (Beijing, China, G1340). The slices from different groups were stained with Weigert’s iron haematoxylin, aniline blue and Ponceau S according to the kit’s instructions. Finally, the slices were photographed using a light microscope (Nikon, Minato, Japan, ×200 magnification).

### Immunohistochemistry assay

The slices from different groups were sealed for 30 min with 3% bovine serum protein (ab64009, Abcam, Cambridge, MA) at 37 °C. After washing with PBS three times, the slides were incubated overnight with anti-α-SMA (ab7817), anti-collagen I (ab88147) and anti-fibronectin (ab2413) antibodies. Then, slices were incubated for 60 min at room temperature with secondary antibody, and incubated for 15 min at room temperature with DAPI. Fields were photographed in each slice using a Leica TCS SP5 microscope (Leica Microsystems, Wetzlar, Germany). All antibodies were purchased from Abcam (Cambridge, MA).

### Enzyme-linked immunosorbent assay (ELISA)

Cytokine concentrations, such as mouse IL-1β, IL-6, TNF-α, urea nitrogen, albumin (ml063132-J; ml002293-J; ml002095-J; ml076479; ml057991, Shanghai Enzyme-linked Biotechnology, Shanghai, China) and creatinine (ab287790, Abcam, Cambridge, MA) in serum or cultured supernatant of fibroblasts were analysed through ELISA in line with specific protocols.

### qRT-PCR analysis

TRIzol reagents (Beyotime Biotechnology, Shanghai, China) were utilized to extract total RNA from renal tissues and fibroblasts. Later, cDNA was prepared from total RNA by TaqMan one-step reverse transcription (Applied Biosystems, Foster City, CA) through reverse transcription. The ABI Prism 7500 system (Applied Biosystems, Foster City, CA) was used for qRT-PCR following specific protocols. We measured the relative mRNA levels of IL-1, IL-6, TNF-α and GAPDH by the 2^−ΔΔCt^ approach. β-Actin served as the endogenous reference. The primers used in this work are shown in Table 1.

**Table 1.** Primer sequences.

Gene name	Primer sequences
β-actin	F: 5′-GCCGGACTCATCGTACTCC-3′
	R: 5′-GTGACGTTGACATCCGTAAAGA-3′
IL-1β	F: 5′-ATCTTTTGGGGTCCGTCAACT-3′
	R: 5′-GCAACTGTTCCTGAACTCAACT-3′
TNF-α	F: 5′-GCTACGACGTGGGCTACAG-3′
	R: 5′-CCCTCACACTCAGATCATCTTCT-3′
IL-6	F: 5′-TTGGTCCTTAGCCACTCCTTC-3′
	R: 5′-T AGGCTGTGATGGTAGCGGA-3′

### Western blot

According to a previous study (Zhou et al. 2022), protein was isolated from renal tissues and fibroblasts, and measured using a BCA kit (Beyotime Biotechnology, Shanghai, China). Protein was extracted using 12% SDS-PAGE and then transferred to PVDF membranes (Millipore, Billerica, MA). Next, membranes were incubated using 5% skimmed milk, followed by overnight incubation with primary antibodies at 4 °C. After rinsing the membranes, they were further incubated for 1 h using HRP-labelled secondary antibody (1:4000, SA00004-10, Proteintech, Shanghai, China) under ambient temperature according to a previous study (Zhou et al. 2022). Finally, an enhanced chemiluminescence kit (ECL, Millipore, Bedford, USA) was utilized to observe protein blots, and ImageJ software (NIH, version 4.3, Bethesda, MD) was used for quantification. All primary antibodies used in this study included anti-Smad2 (1:2000, 12570-1-AP, Proteintech, Shanghai, China), anti-p-Smad2 (1:2000, ab188334, Abcam, Cambridge, MA), anti-Smad3 (1:2000, 25494-1-AP, Proteintech, Shanghai, China), anti-p-Smad3 (1:2000, ab52903, Abcam, Cambridge, MA), anti-α-SMA (1:2000, ab7817, Abcam, Cambridge, MA), anti-Collagen I (1:2000, 14695-1-AP, Proteintech, Shanghai, China), anti-fibronectin (1:2000, 15613-1-AP, Proteintech, Shanghai, China) and anti-GAPDH (1:5000, 60004-1-Ig, Proteintech, Shanghai, China), with β-actin being the endogenous control.

### Statistical analysis

Data were analysed using GraphPad Prism 5.0 (La Jolla, CA) and are presented as the mean ± SD. Differences between groups were compared by one-way ANOVA and Tukey’s post hoc test. *p* < 0.05 indicated statistical significance.

## Results

### Effects of baicalin on renal histological changes in UUO mice

**Figure 1.**
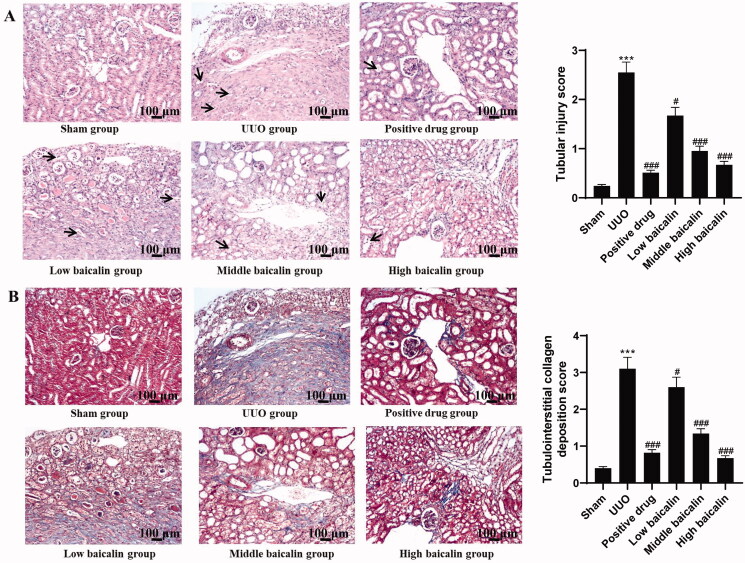
Effects of baicalin on renal histological changes in UUO mice. (A) H&E assay was performed to evaluate the effects of baicalin on renal histological changes in UUO mice (100× magnification, bar = 100 μm). (B) Masson staining assay was performed to assess the effects of baicalin on collagen deposition in renal tissues (100× magnification, bar = 100 μm). ***p < 0.001 vs. sham group, ^#^*p* < 0.05, ^###^*p* < 0.001 vs. UUO group.

**Figure 2.**
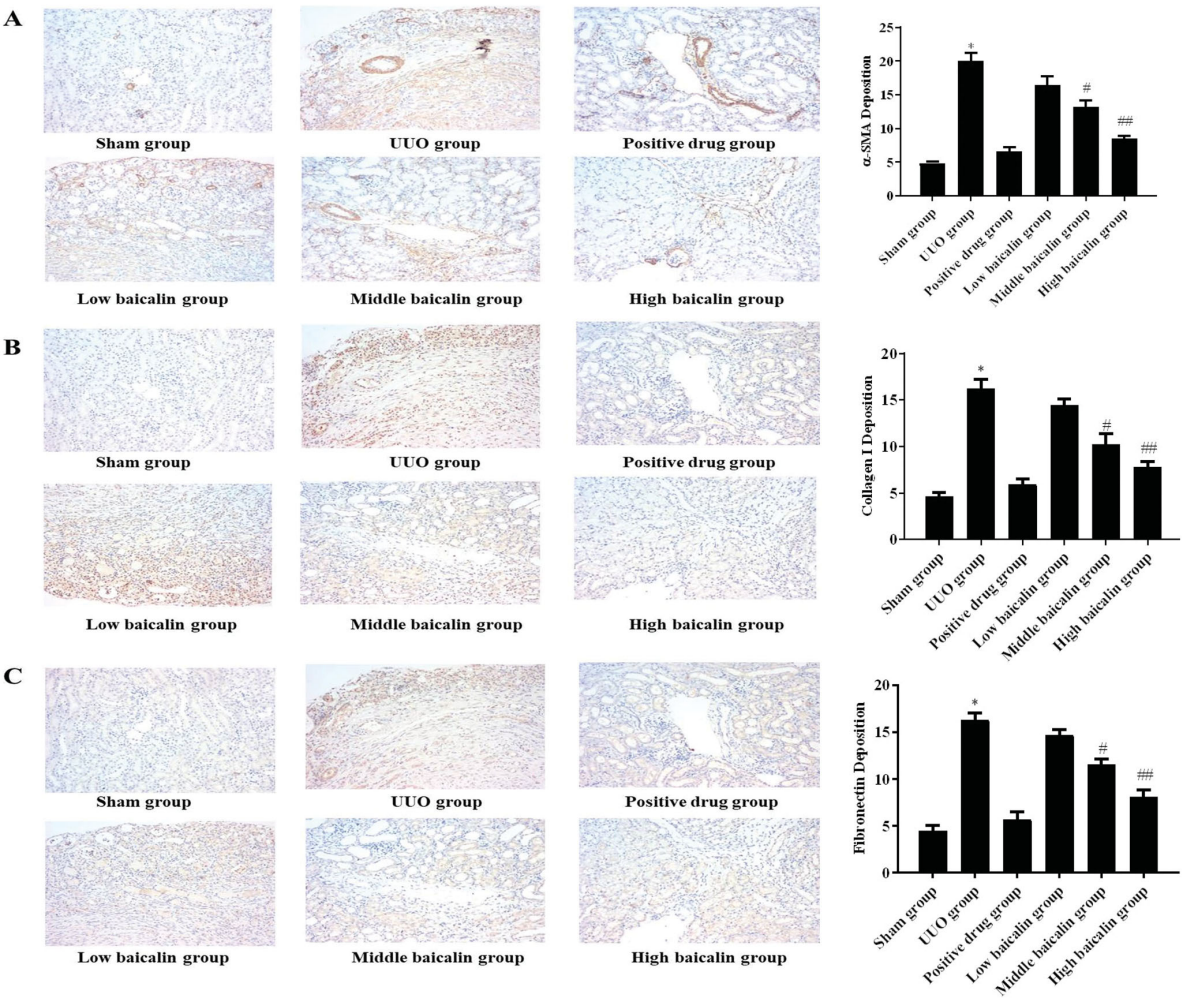
Effects of baicalin on the expression of proteins, including α-SMA, collagen I and fibronectin, in UUO mice. (A–C) Immunohistochemical staining was performed to determine the effects on the expression of (A) α-SMA, (B) collagen I and (C) fibronectin in UUO mice. *p < 0.05 vs. sham group, ^#^*p* < 0.05, ^##^*p* < 0.01 vs. UUO group.

A mouse UUO model was constructed, and different doses of baicalin were administered. The H&E assay data showed that mice in the sham group showed normal glomerular structure. The tubular epithelial cells had abundant cytoplasm, a small lumen and a small interstitial area, and no renal tubule expansion, atrophy or inflammatory cell infiltration was found. After UUO modelling, the renal cortex became thinner, the renal tubules expanded, vacuolar degeneration of tubular epithelial cells appeared, and exfoliated epithelial cells were scattered in the lumen. Baicalin notably improved the degree of renal pathological changes in UUO mice (Figure 1(A)). The Masson assay was performed and the results indicated that in the sham group, the boundary between cortex and medulla of renal tissues was clear, the structure of renal tubules, glomeruli and renal interstitium was not abnormal, and only part of the renal tubular basement membranes had linear blue stained collagen tissues. In the UUO model group, the renal tubules were dilated and obviously atrophied, the renal interstitium was widened, and the area of blue stained reticular fibres in the stroma was larger. As expected, compared with the model group, baicalin treatment improved the renal histological changes of UUO mice (Figure 1(B)). Moreover, immunohistochemistry was used to assess α-SMA, collagen I and fibronectin expression. Relative to the sham group, α-SMA, collagen I and fibronectin were increased by approximately threefold, in the UUO group, while baicalin remarkably inhibited the expression of these proteins by 15–60%, 12–50% and 12–50%, respectively, as shown in Figure 2(A–C). These data suggested that baicalin improved the renal histological changes in UUO mice.

### Effects of baicalin on inflammatory responses of UUO mice

**Figure 3.**
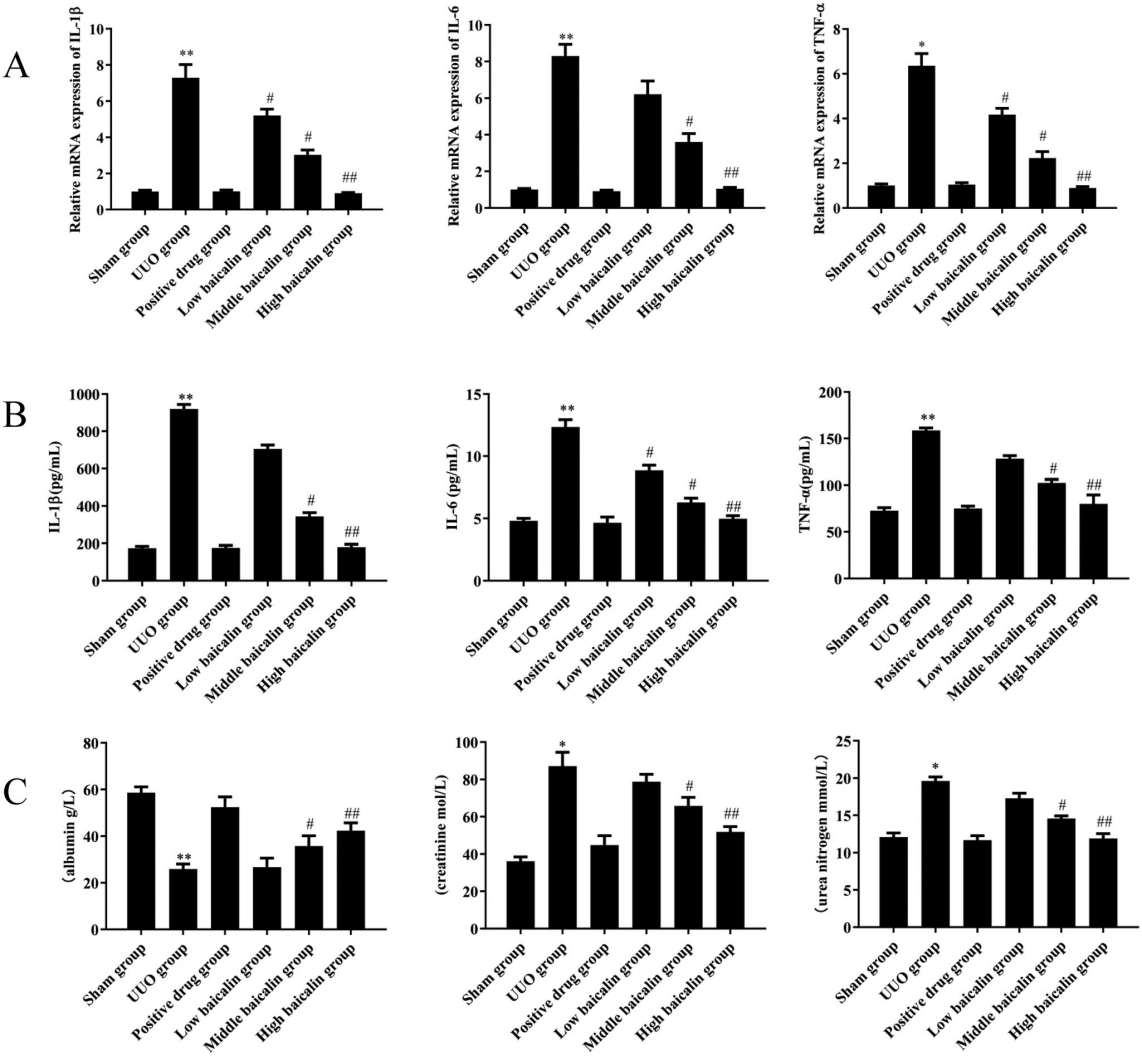
Effects of baicalin on inflammatory responses of UUO mice. (A) qRT-PCR assays were performed to evaluate the effects of baicalin on the mRNA expression of IL-1β, IL-6 and TNF-α in UUO mice. (B) ELISA was performed to assess the effects of baicalin on the expression of IL-1β, IL-6 and TNF-α in UUO mice. (C) ELISA was performed to determine the effects of baicalin on the contents of urea nitrogen, creatinine and albumin in UUO mice. *n* = 6. *p < 0.05, **p < 0.01 vs. the sham group, ^#^*p* < 0.05, ^##^*p* < 0.01 vs. the UUO group.

Inflammatory responses play an important role in the progression of RIF (Kim et al. 2014). qRT-PCR and ELISA were used to determine the levels of, IL-1β, IL-6 and TNF-α in serum. As shown in Figure 3(A,B), compared to the sham group, serum IL-1β, IL-6 and TNF-α expression was significantly upregulated by 4-fold, 1.4-fold and 1.2-fold, respectively, in the UUO group, while baicalin treatment markedly decreased the expression levels of IL-1β, IL-6 and TNF-α by 81.4%, 59.5% and 49.2%, respectively, in a dose-dependent manner. The middle baicalin and high dose baicalin groups exhibited the most significant difference compared to the sham group.

Moreover, the contents of urea nitrogen, creatinine and albumin were also detected. The contents of urea nitrogen, creatinine and albumin were obviously increased in the serum of UUO mice, whereas baicalin treatment significantly reduced the production of urea nitrogen, and creatinine by 38.8% and 40.9% at most and increased that of albumin by 57% at most in a dose-dependent manner (Figure 3(C)). These data suggested that baicalin inhibited the inflammatory responses of UUO mice.

### Effects of baicalin on the TGF-β/Smad pathway in UUO mice

**Figure 4.**
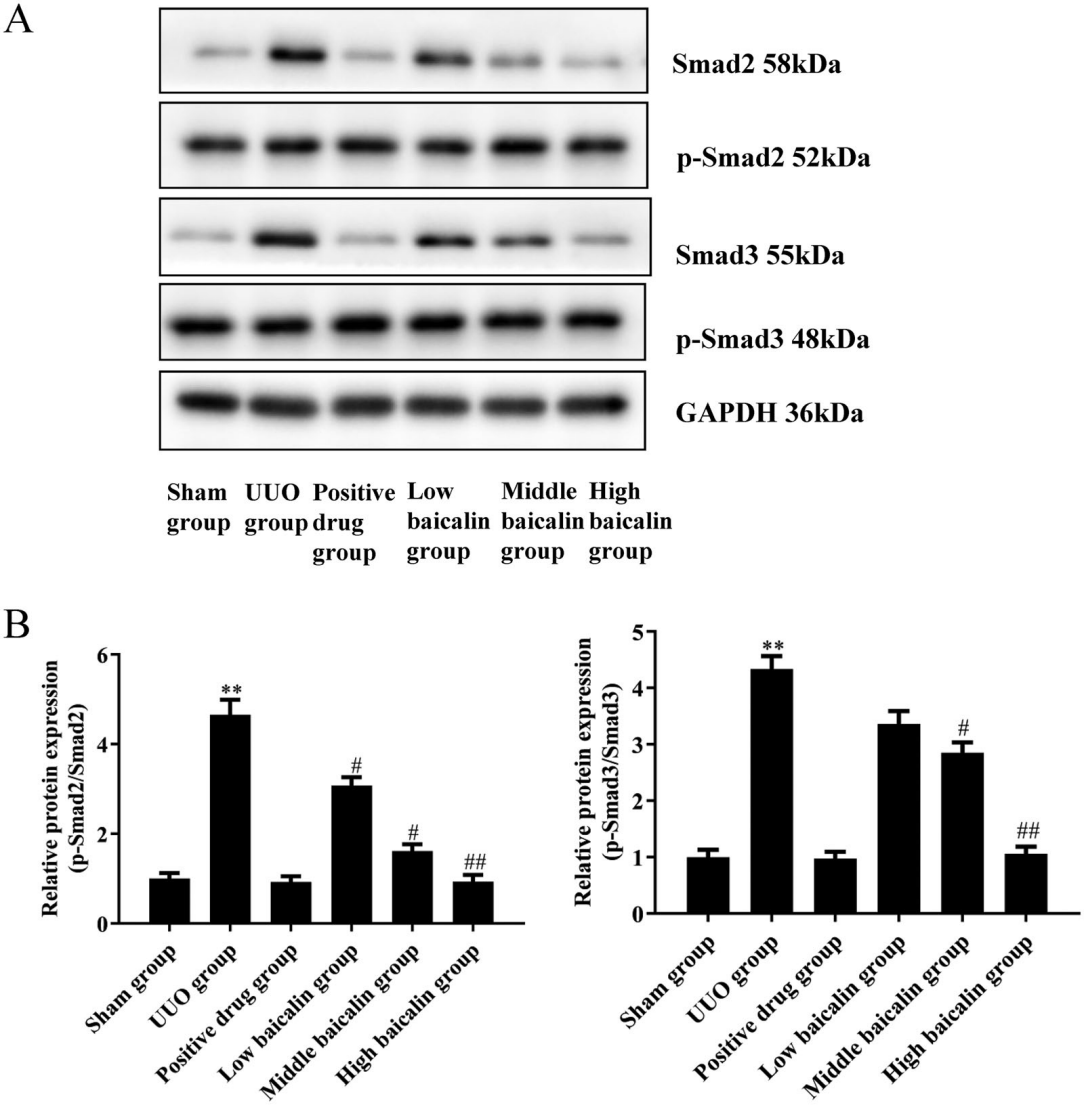
Effects of baicalin on the TGF-β/Smad signalling pathway in UUO mice. (A, B) Western blot assays were performed to evaluate the effects of baicalin on the expression of the TGF-β/Smad signalling pathway in UUO mice. *n* = 6. **p < 0.01 vs. the sham group, ^#^*p* < 0.05, ^##^*p* < 0.01 vs. the UUO group.

Activation of TGF-β/Smad exerts a vital effect on the progression of RIF (Chen L et al. 2018). Smad proteins act as intracellular signalling mediators of TGF-β. Therefore, the protein expression of Smad2, and Smad3, and the levels of phosphorylation of Smad2 and Smad3, as well as TGF-β, were investigated by western blotting. The results showed that compared with the sham group, the UUO group showed obviously increased levels of phosphorylated Smad2 and Smad3. In addition, the UUO group exhibited higher levels of TGF-β protein expression. Baicalin treatment decreased the activated phosphorylation of Smad2 (79.4% decrease) and Smad3 (75.4% decrease) in a dose-dependent manner. Similarly, the levels of TGF-β protein expression were reduced (66.3% decrease) with baicalin treatment in a dose-dependent manner. These findings indicate that baicalin inhibited the activation of the TGF-β/Smad pathway (Figure 4(A,B)).

### Effects of baicalin on ECM expression and inflammatory factor secretion in mouse primary fibroblasts

To further investigate the role of baicalin in RIF, mouse primary fibroblasts were isolated and treated with TGF-β1 and/or baicalin at concentrations of 50, 100 and 150 μmol/L (low baicalin group, medium baicalin group and high baicalin group, respectively).

**Figure 5.**
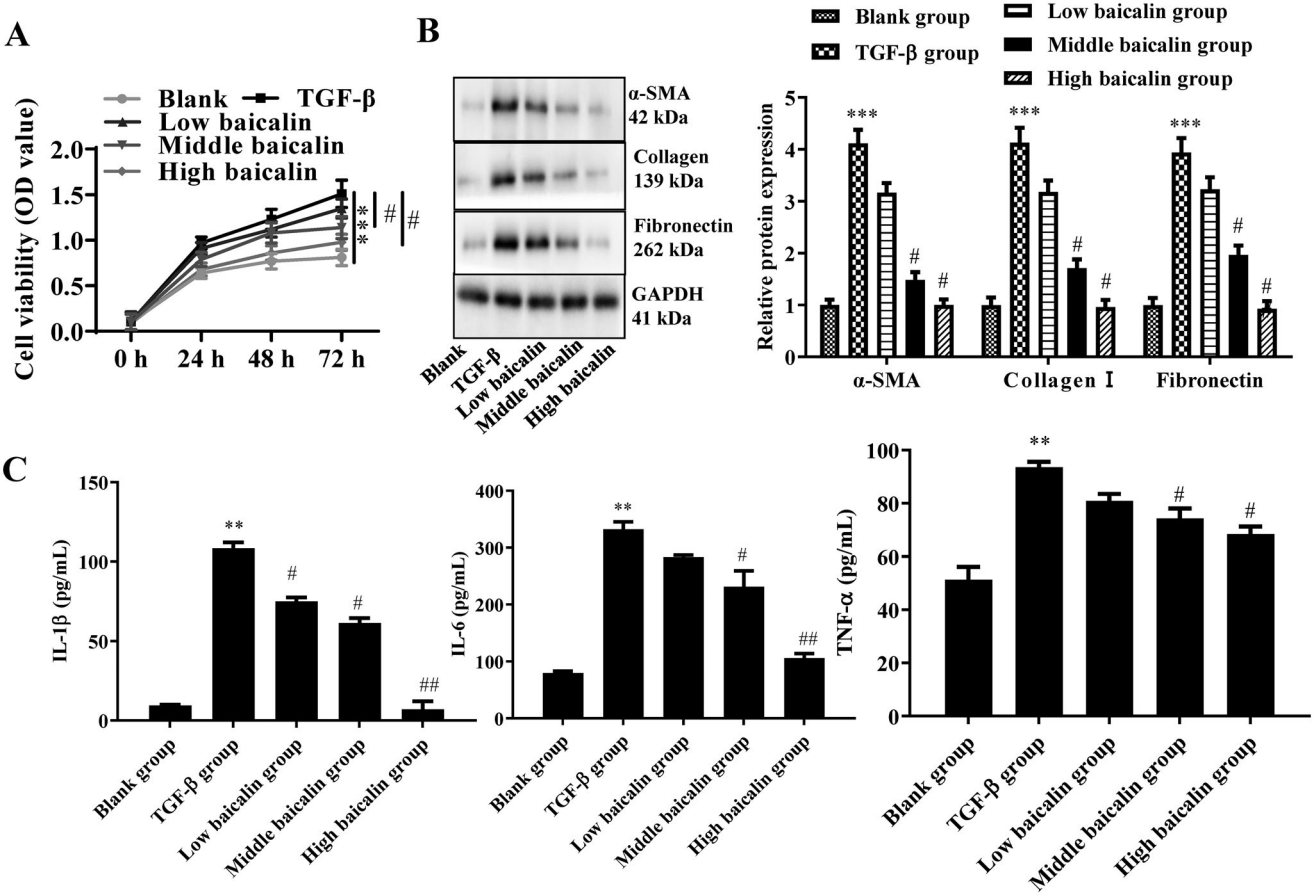
Effects of baicalin on ECM expression and inflammatory factor secretion in mouse primary fibroblasts. (A, B) Western blot assays were performed to determine the effects of baicalin on the expression of fibrosis-related proteins in TGF-β1-treated fibroblasts. (C) ELISA assay was performed to assess the effects of baicalin on the expression of IL-1β, IL-6 and TNF-α in TGF-β1-treated fibroblasts. *n* = 6. **p < 0.01, ***p < 0.001 vs. the blank group, ^#^*p* < 0.05, ^##^*p* < 0.01 vs. the TGF-β1 group.

The expression levels of α-SMA, collagen I and fibronectin were then assessed in the supernatant of primary fibroblasts using western blot analysis. The data in Figure 5(A,B) show that α-SMA, collagen I and fibronectin were highly expressed in fibroblasts treated with TGF-β1, while baicalin remarkably inhibited the expression of these proteins within TGF-β1-treated fibroblasts by 22.6–75.4% (α-SMA), 24–77.6% (collagen I) and 18.5–76.3% (fibronectin). In addition, ELISAs were carried out to determine the levels of inflammatory factors (IL-1β, IL-6 and TNF-α). As illustrated in Figure 5(C), the expression levels of IL-1β, IL-6 and TNF-α were notably upregulated, while baicalin treatment partially suppressed the expression levels of IL-1β, IL-6 and TNF-α. Specifically, the expression of IL-1β was reduced by 31.9% with a low dose of baicalin, 43.8% with a middle dose and 93.1% with a high dose. The expression of IL-6 was reduced by 14% with a low dose, 30.2% with a middle dose and 68% with a high dose. The expression of TNF-α was reduced by 13.5% with the low dose, 21% with the middle dose and 26.6% with the high dose. These data suggested that baicalin inhibited the expression of ECM and the secretion of inflammatory factors in IL-1β-treated fibroblasts.

### Effects of baicalin on fibrosis of mouse primary fibroblasts mediated by the TGF-β/Smad pathway

**Figure 6.**
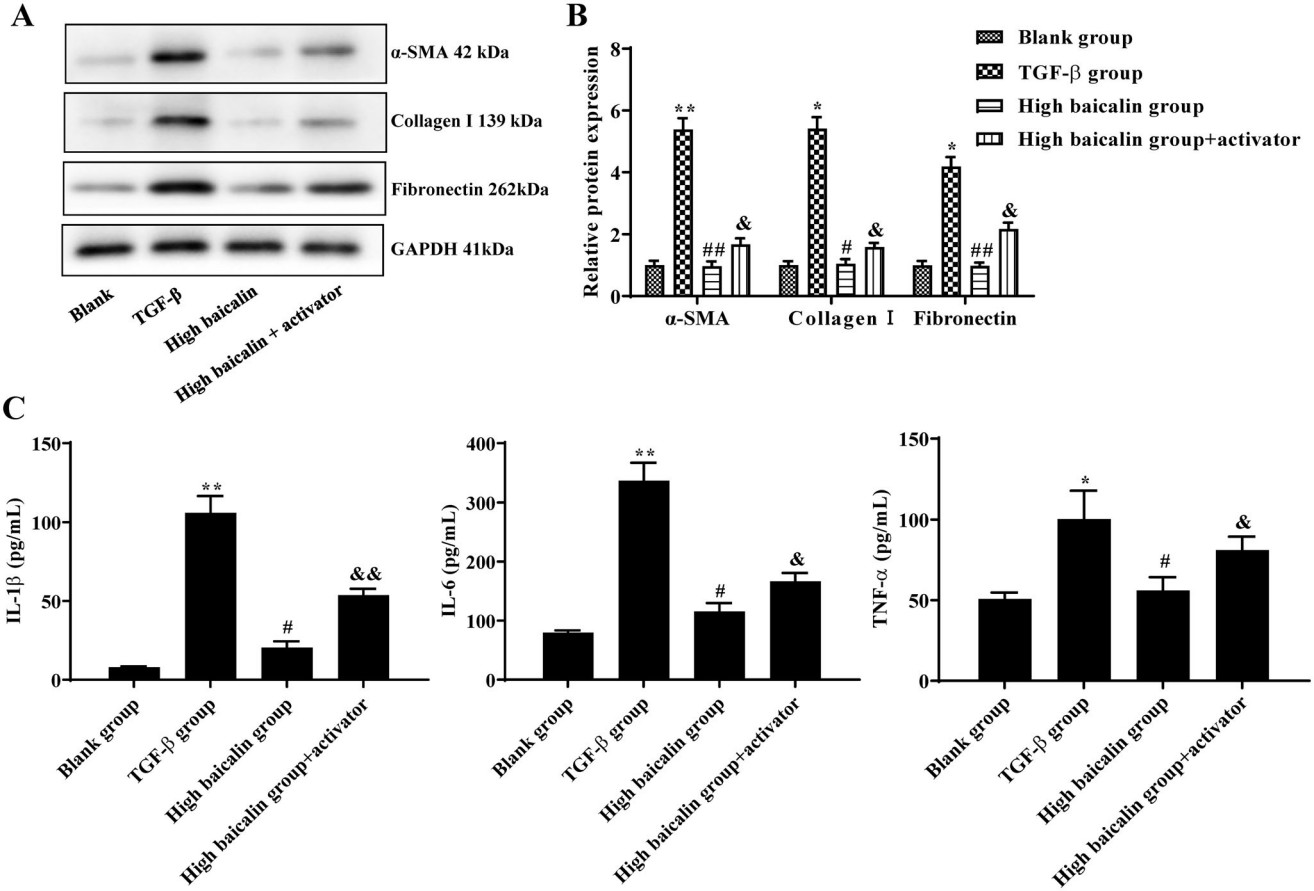
Effects of baicalin on fibrosis of mouse primary fibroblasts mediated by the TGF-β/Smad signalling pathway. (A, B) Western blot assays were performed to determine the effects of baicalin or activator on the expression of fibrosis-related proteins in TGF-β1-treated fibroblasts. (C) ELISA was performed to assess the effects of baicalin or activator on the expression of IL-1β, IL-6 and TNF-α in TGF-β1-treated fibroblasts. *n* = 6. *p < 0.05, **p < 0.01 vs. the blank group, ^#^*p* < 0.05, ^##^*p* < 0.01 vs. the TGF-β1 group, ^&^*p* < 0.05, ^&&^*p* < 0.01 vs. the baicalin group.

To investigate whether baicalin protected against RIF by regulating the TGF-β/Smad pathway, the TGF-β/Smad pathway activator (SRI-011381 hydrochloride) was cotreated with baicalin (150 μmol/L; high baicalin group) in mouse primary fibroblasts. Western blotting was used to determine the protein levels of α-SMA, collagen I and fibronectin in fibroblasts, and the results showed that the TGF-β group exhibited higher levels of α-SMA, collagen I, and fibronectin; however, baicalin showed significantly lower levels of α-SMA, collagen I and fibronectin. When the mouse primary fibroblasts were treated with baicalin + TGF-β/Smad activator, it was found that the baicalin + TGF-β/Smad activator group had reduced levels of α-SMA, collagen I and fibronectin compared with the TGF-β group (Figure 6(A,B)). Next, ELISA was adopted to detect the expression of inflammatory factors in the supernatant of TGF-β-treated fibroblasts. The IL-1β, IL-6 and TNF-α levels were increased in the TGF-β group; however, the baicalin + TGF-β/Smad activator group showed decreased levels of IL-1β, IL-6 and TNF-α compared to the TGF-β group (Figure 6(C)). Baicalin cotreatment with an activator reversed the effects of baicalin on fibrosis-related protein levels and inflammatory factor expression in TGF-treated fibroblasts. These data suggested that baicalin protected against fibrosis in mouse primary fibroblasts partially through regulating the TGF-β/Smad signalling pathway.

## Discussion

CKD has become a common public health problem worldwide, causing the suffering of patients and a huge economic burden to families and society (Yang et al. 2018). Moreover, the long-term use of cytotoxic drugs to cure CKD results in immunosuppression and many other side effects (Goumenos et al. 2007; Kędzierska et al. 2011). In addition, some new drugs are too expensive for patients to afford, resulting in treatment interruption. Therefore, an increasing number of researchers have begun to pay attention to the development of Chinese herbal medicine or extract effective components to treat kidney disease, which has become one of the important research trends in recent years (Xia et al. 2016). *Poria cocos* Wolf (Polyporaceae), a well-known kind of TCM, contains many compounds that act as renin–angiotensin system inhibitors through the TGF-β1/Smad pathway (Wang M, Chen, Chen, Cao, et al. 2018; Wang M, Chen, Chen, Liu, et al. 2018; Chen DQ et al. 2020). Studies have also demonstrated that two species of Alismataceae, also known as efficacious CTMs, have protective functions by blocking TGF-β1 (Chen H et al. 2018, 2020).

Baicalin is a flavonoid extracted from the dried root of *Scutellaria baicalensis* with many pharmacological effects. Animal experiments and clinical studies have confirmed that baicalin has antifibrosis and organ protection effects (Xiao et al. 2018). However, the precise role and potential mechanisms have yet to be revealed. In the current study, UUO mouse models were established and treated with varying doses of baicalin. Baicalin treatment clearly ameliorated RIF in UUO mice, confirming the protective effects of baicalin on RIF. Fibroblasts are intrinsic cells in the renal interstitium that transform into myofibroblasts under the stimulation of TGF-β (Sun et al. 2016). Recent studies have confirmed that myofibroblasts are associated with RIF and are the key effector cells. The phenotypic transformation of fibroblasts and renal cells to myofibroblasts is an important mechanism leading to RIF. Interestingly, baicalin provided significant protection against TGF-β1-induced myofibroblasts.

The ECM is a component of the stroma and epithelial vascular matrix, including collagen fibres (type I, III, IV), noncollagen glycoproteins (such as fibronectin, laminin), proteoglycans, elastin and aminoglycan, which are involved in the synthesis and degradation of the ECM, further playing a key role in RIF (Bülow and Boor 2019). EMT is the process by which mature epithelial cells lose their epithelial characteristics and acquire immature mesenchymal characteristics. Additionally, EMT plays a role in a variety of physiological and pathological processes, including embryonic development, tumour metastasis, wound healing and cell migration (Lovisa et al. 2015). In the process of RIF, regardless of the primary disease, α-SMA-positive myofibroblasts are the main cells that lead to excessive deposition of ECM in the renal interstitium under pathological conditions (Chen Y et al. 2019). Therefore, in our study, α-SMA and ECM-related proteins (collagen I and fibronectin) were detected through IHC and western blot assays. The results showed that baicalin significantly reduced these expression levels. Meanwhile, both in *in vivo* and *in vitro* assays, inflammatory factors (IL-1β, IL-6 and TNF-α) and renal function indexes (urea nitrogen, creatinine and albumin) were detected to further determine the change in renal injury. Inflammatory factors are associated with RIF by controlling the production and release of profibrotic cytokines and growth factors (Meng et al. 2014). Our results showed that the group treated with baicalin tended to have the most similar expression to the blank group, which demonstrated that baicalin protects against RIF based on histological and inflammatory results.

**Figure 7.**
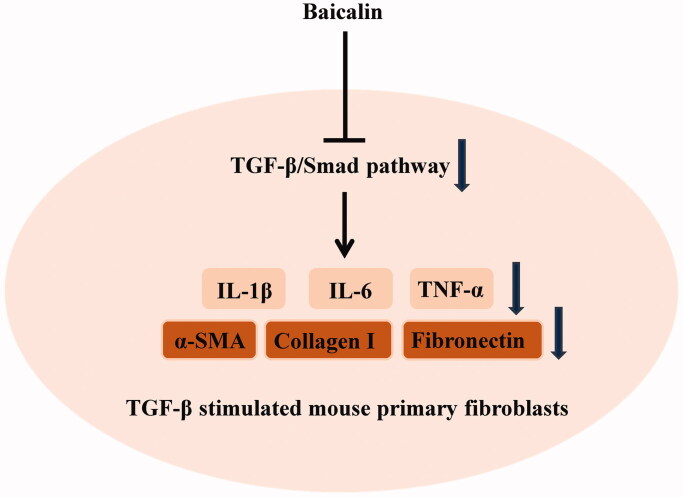
Baicalin exhibited negative effects on the inflammatory response and ECM synthesis by blocking the TGF-β/Smad signalling pathway in TGF-β-stimulated mouse primary fibroblasts.

TGF-β1 has been reported to exert key effects on RIF (Ding et al. 2017) and can promote renal fibrosis by stimulating the production of ECM and inhibiting its degradation (Lan 2011). The TGF-β1/Smad pathway has a critical role in renal fibrosis, and Smad2 and Smad3 are two critical downstream mediators of TGF-β1's biological effects (Xu J et al. 2018). In the state of disease, TGF-β1 binds to TβRII and activates TGF-β1 receptor I kinase, thus promoting Smad2 and Smad3 phosphorylation. Phosphorylated Smad2/Smad3 (p-Smad2/3) combines with Smad4 to form the Smad complex, and then translocates to the nucleus to regulate the transcription of corresponding target genes, which is a key step in the activation of the TGF-β1/Smad signalling pathway (Xu P et al. 2012). TGF-β1 and p-Smad2/3 protein expression in the renal tissues of UUO rats increases with time of obstruction, and the degree of RIF is also aggravated (Chen J and Li 2018). Moreover, it has been suggested that increased TGF-1 activation activates the Smad signal transduction pathway and promotes p-Smad2 and p-Smad3 expression (Wang L et al. 2015). In our study, increased levels of p-Smad2 and p-Smad3 were observed in UUO mice and in TGF-β1-treated myofibroblasts. In contrast, baicalin treatment significantly decreased p-Smad2, p-Smad3 and TGF-β protein expression. Our data suggested that baicalin treatment inhibited the activation of the TGF-β/Smad pathway both in UUO mice and in TGF-β1-treated myofibroblasts (Figure 7). To further verify the role of the TGF-β/Smad pathway in RIF, a TGF-β/Smad signalling pathway activator was used, and we found that baicalin cotreatment with the activator reversed the effects of baicalin on fibrosis-related protein levels and inflammatory factor expression in TGF-β-treated fibroblasts.

## Conclusions

Baicalin exhibited potential therapeutic properties against RIF by inhibiting the TGF-β/Smad signalling pathway. Therefore, our study provides evidence that baicalin may emerge as a therapeutic option for RIF treatment.

## Author contributions

Hui Wang and Lizhu Zhang were involved in the conception and design of this study. Hui Wang and Qingtao Jiang performed the data analysis and interpreted the results. Hui Wang and Qingtao Jiang prepared the first draft of the manuscript. Lizhu Zhang critically revised the revision of the manuscript. Lizhu Zhang supervised the study.
